# Sequence Specific Binding of Beta Carboline Alkaloid Harmalol with Deoxyribonucleotides: Binding Heterogeneity, Conformational, Thermodynamic and Cytotoxic Aspects

**DOI:** 10.1371/journal.pone.0108022

**Published:** 2014-09-23

**Authors:** Sarita Sarkar, Prateek Pandya, Kakali Bhadra

**Affiliations:** Department of Zoology, University of Kalyani, Nadia, West Bengal, India; St. Georges University of London, United Kingdom

## Abstract

**Background:**

Base dependent binding of the cytotoxic alkaloid harmalol to four synthetic polynucleotides, poly(dA).poly(dT), poly(dA-dT).poly(dA-dT), poly(dG).poly(dC) and poly(dG-dC).poly(dG-dC) was examined by various photophysical and calorimetric studies, and molecular docking.

**Methodology/Principal Findings:**

Binding data obtained from absorbance according to neighbor exclusion model indicated that the binding constant decreased in the order poly(dG-dC).poly(dG-dC)>poly(dA-dT).poly(dA-dT)>poly(dA).poly(dT)>poly(dG).poly(dC). The same trend was shown by the competition dialysis, change in fluorescence steady state intensity, stabilization against thermal denaturation, increase in the specific viscosity and perturbations in circular dichroism spectra. Among the polynucleotides, poly(dA).poly(dT) and poly(dG).poly(dC) showed positive cooperativity where as poly(dG-dC).poly(dG-dC) and poly(dA-dT).poly(dA-dT) showed non cooperative binding. Isothermal calorimetric data on the other hand showed enthalpy driven exothermic binding with a hydrophobic contribution to the binding Gibbs energy with poly(dG-dC).poly(dG-dC), and poly(dA-dT).poly(dA-dT) where as harmalol with poly(dA).poly(dT) showed entropy driven endothermic binding and with poly(dG).poly(dC) it was reported to be entropy driven exothermic binding. The study also tested the *in vitro* chemotherapeutic potential of harmalol in HeLa, MDA-MB-231, A549, and HepG2 cell line by MTT assay.

**Conclusions/Significance:**

Studies unequivocally established that harmalol binds strongly with hetero GC polymer by mechanism of intercalation where the alkaloid resists complete overlap to the DNA base pairs inside the intercalation cavity and showed maximum cytotoxicity on HepG2 with IC_50_ value of 14 µM. The results contribute to the understanding of binding, specificity, energetic, cytotoxicity and docking of harmalol-DNA complexation that will guide synthetic efforts of medicinal chemists for developing better therapeutic agents.

## Introduction

Sequence specific binding of small molecules to DNA continues to attract considerable attention for developing effective therapeutic agents for control of gene expression [1]–[5]. Functionally, deoxyribonucleic acid serves as the repository of the genetic information of the cell, hence it is thought to be the cellular target of many therapeutic molecules.

Alkaloids represent a group of interesting natural small molecules abundantly available in nature. Many, if not all, of the alkaloids isolated so far have been shown to have remarkable medicinal applications that may be exploited effectively for the betterment of the mankind. Beta–carboline alkaloids are a large group of natural and synthetic indole alkaloids with different degrees of aromaticity [6]. Beta carbolines were first isolated from *Peganum harmala* (Zygophillaceae), which were used as a traditional herbal drug in the Middle East and North Africa [7]. Beta carboline alkaloids have been reported to have several pharmacological, neurophysiological and biochemical activities. They include inhibition of cytochrome P450 [8], inhibition of monoamine oxidase [9], binding to several serotonin, benzodiazepines and dopamine receptors [10] and inhibition of DNA topoisomerase activities [11]. Toxic and genotoxic effects of beta carboline alkaloids have been reported in both prokaryotic and eukaryotic cells. Some of the mutagenic and carcinogenic effects of various carboline alkaloids have been related to their ability to intercalate into DNA [12], [13] leading to altered DNA replication fidelity and enzymatic activities in DNA repair processes [11], [14]. Extracts of *P. harmala* seeds tested *in vitro* on mice skin carcinoma and sarcoma cell lines significantly reduced cell proliferation [15]. Harman and norharman induced apoptosis and necrosis in Human neuroblastoma SH-SY5Y cells, [16].

**Figure 1:**
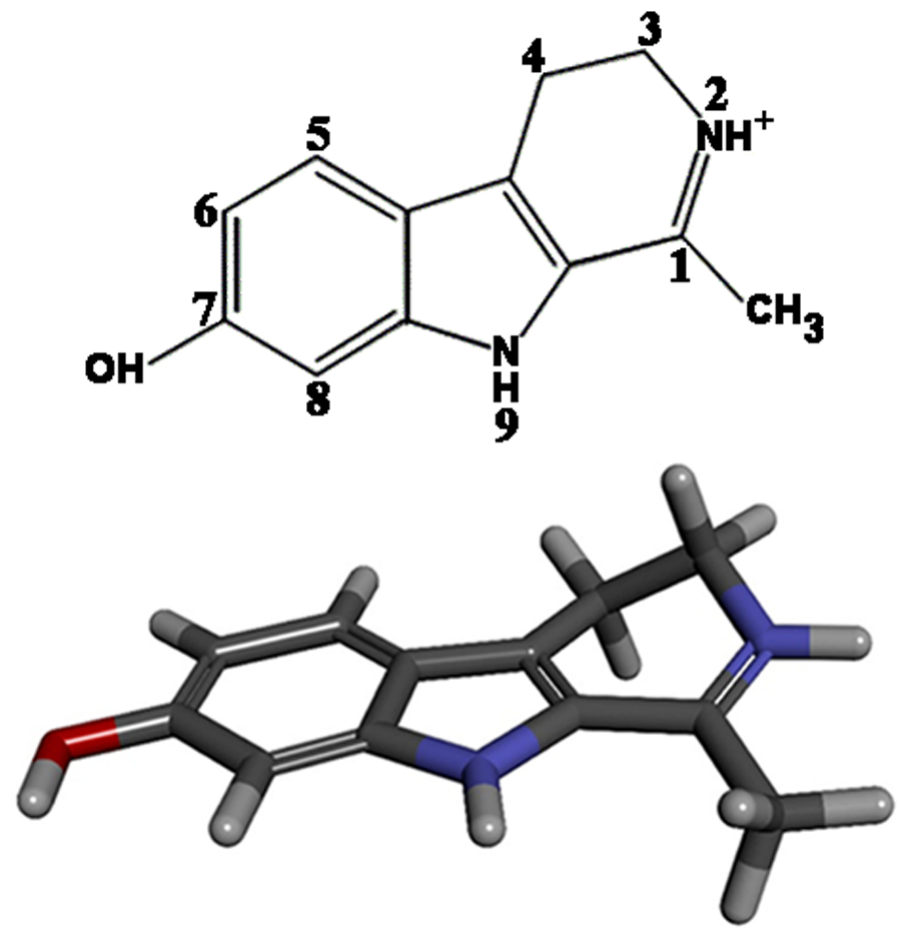
Chemical structure of harmalol in 2 and 3D view.

Though the interaction of beta carboline alkaloids with DNA [17]–[24] has been studied earlier no detailed information on the sequence specificity and thermodynamic aspects of the interaction has been reported. In order to understand the anticancer/biological properties of the beta caroline alkaloids detailed knowledge of the mode, mechanism, energetics and specificity of their interaction with nucleic acid is necessary. Therefore in this paper we studied the interaction of harmalol (3,4-dihydro-1-methyl-9H-pyrido[3,4-b] (Fig. 1), one of the most important representative of this group of alkaloids, with four synthetic sequence specific polynucleotides.We present new insights into a structural aspects of the interaction in terms of cooperativity/non cooperativity base pair heterogeneity, thermodynamics of the interaction and binding model through molecular docking studies. The chemotherapeutic potential in terms of its response to different human cancer cell lines was also studied.

## Results and Discussion

### Competition dialysis assay

**Figure 2:**
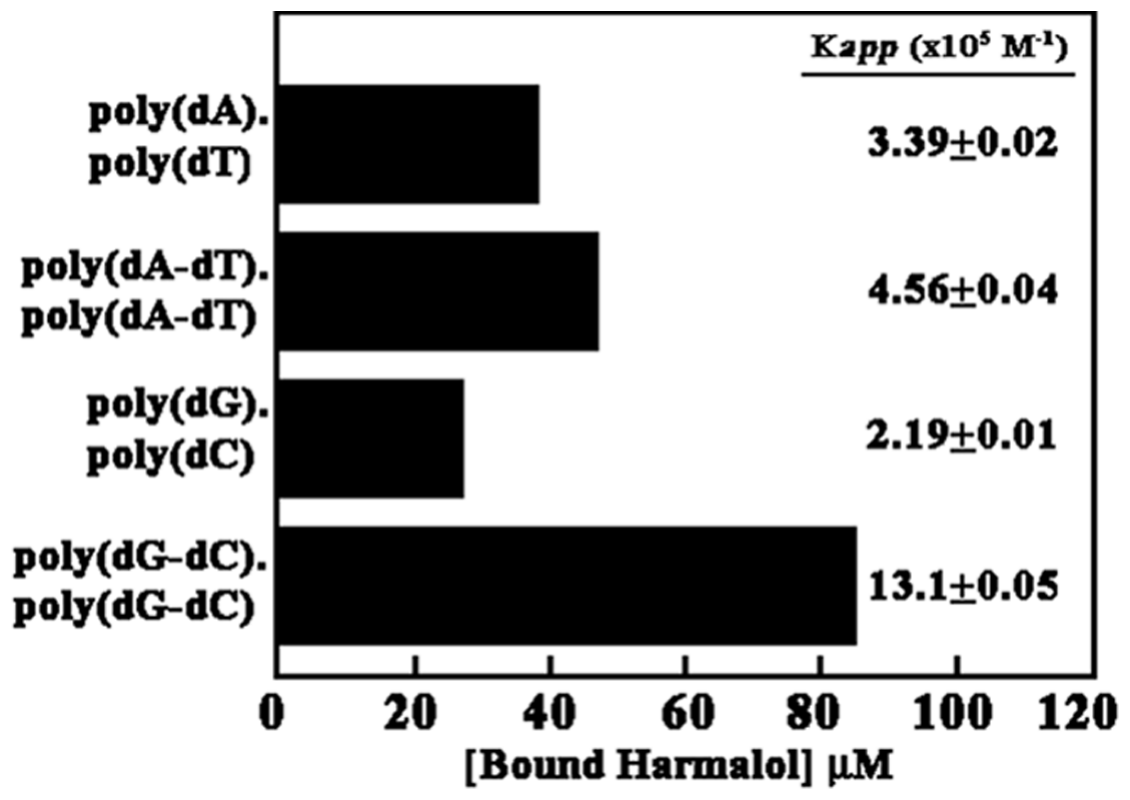
Results of competition dialysis experiment in 15 mM CP buffer, pH 6.8 at 25±0.5°C. The concentration of harmalol bound to each polynucleotide sample is shown as a bar graph. The data given are average of three independent experiments under identical conditions.

Figure 2 shows the result from competition dialysis assay of the four polynucleotide samples (of 150 µM concentration) *viz.* poly(dA).poly(dT), poly(dA-dT).poly(dA-dT), poly(dG).poly(dC) and poly(dG-dC).poly(dG-dC), dialyzed against 1 µM of harmalol presented as bar graphs in which concentration of alkaloid bound to each of the polynucleotide sample is plotted. The competition dialysis assay is a new, effective and powerful tool based on fundamental thermodynamic principle of equilibrium dialysis for the discovery of ligand that can bind to nucleic acids with structural and sequence selectivity [25], [26]. The striking result that emerges from this experiment is the pronounced binding of harmalol to poly(dG-dC).poly(dG-dC) followed by poly(dA-dT).poly(dA-dT). Binding of the alkaloid is found to be significantly weak with both the homo polynucleotide *viz.* poly(dG).poly(dC) and poly(dA).poly(dT). From these data *K_app_* was calculated and the values were found to be 13.10±0.07×10^5^ M^−1^, 4.56±0.04×10^5^ M^−1^, 2.19±0.01×10^5^ M^−1^ and 3.39±0.02×10^5^ M^−1^, respectively, for poly(dG-dC).poly(dG-dC), poly(dA-dT).poly(dA-dT), poly(dG).poly(dC) and poly(dA).poly(dT). The results indicate the affinity of harmalol to be maximum with poly(dG-dC).poly(dG-dC) and to the other polymers it varied in the order of poly(dA-dT).poly(dA-dT)>poly(dA).poly(dT)>poly(dG).poly(dC).

### Spectrophotometric studies and analysis of binding affinity

**Figure 3:**
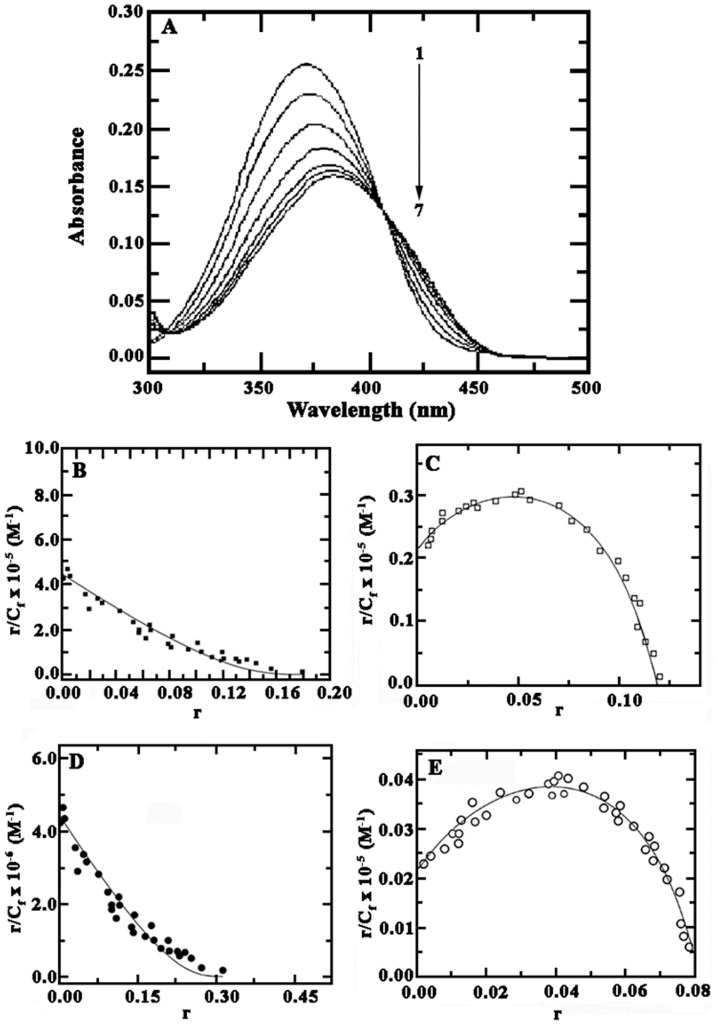
(A) (Top panel) A representative figure showing the changes in the absorption spectra of harmalol upon titration with poly(dG-dC).poly(dG-dC) in 15 mM CP buffer, pH 6.8 at 25±0.5°C. Curve (1) denote absorption spectrum of harmalol (10.0 µM) treated with 5.16, 10.5, 20.2, 30.50, 35.0 and 50.0 µM (curves 2–7) of poly(dG-dC).poly(dG-dC), respectively. (Bottom panel) Scatchard plots derived from absorbance spectral data for the binding of harmalol to 20 µM of (B) hetero AT, (C) homo AT, (D) hetero GC and (E) homo GC polynucleotides. The data was fit to both cooperative and non cooperative binding model derived from McGhee-von Hippel equation [68]. The best fit values of *K_b_*, *n* and *ω* are presented in Table 1.

The binding between harmalol and polynucleotides was further examined by rigorous UV-vis absorption titrations. The characteristic UV- visible spectrum of the alkaloid exhibits maxima at 207, 258 and 371 nm in the range of 200–550 nm [24]. A representative case illustrating the absorption spectral changes of the alkaloid (fixed concentration) resulting from the interaction of varied concentration of poly(dG-dC).poly(dG-dC) (here after hetero GC) with harmalol (10 µM) is depicted in figure 3A. On titration with hetero GC, the spectra exhibit a characteristic hypochromic effect of 44% and a bathochromic shift of 13 nm until saturation was reached at P/D (nucleotide phosphate/alkaloid molar ratio) 5.0. The hypochromic and bathochromic effects essentially indicate strong intermolecular interaction involving effective overlap of the π electron cloud of harmalol with the nucleotide bases and are speculative of intercalative ligand–DNA complexation. The spectra show a clear isosbestic point at 407 nm indicating a clear equilibrium between free and DNA bound form of the structure. These remarkable spectral changes disclosed π-π-stacking interactions between the chromophore of this molecule and the DNA. Similar results were also observed with poly(dA-dT).poly(dA-dT) (here after hetero AT), poly(dG).poly(dC) (here after homo GC) and poly(dA).poly(dT) (here after homo AT), but the extent of spectral changes were different (figures not shown) being markedly higher with hetero GC followed by hetero AT and least with homo AT and GC polymers. The values of the hypochromic effect and bathochromic shift of all the four polynucleotides are presented in Table 1. The molar extinction coefficients of the fully bound alkaloid at the wavelength maxima and at the isosbestic points were determined by the above titration. In Fig. 3 A (upper panel) spectrum 1 denotes the free ligand and spectra 8 and 9 denote the fully bound ligand, respectively. From this titration the change in molar extinction coefficient Δε was calculated. The spectrophotometric titration data of the increasing concentration of alkaloid to a fixed concentration of DNA was employed to evaluate the binding affinity of the alkaloid using Scat chard plots. The binding spectra of harmalol to all the four synthetic DNAs are illustrated in Fig. 3 B–E (lower panel). Scatchard plots show that harmalol binds both in a cooperative or non-cooperative manner depending on the base composition and sequence of base pairs. Positive cooperative binding was observed with poly(dA).poly(dT) and poly(dG).poly(dC), while non-cooperative binding was seen with poly(dA-dT).poly(dA-dT) and poly(dG-dC).poly(dG-dC). The quantitative data of binding parameters calculated from fitting of these plots to the appropriate McGhee-Von Hippel equation 1 and 2 are presented in Table 1. Thus, comparing the data on the hypochromic effect, bathochromic shift and the binding constant calculated from the fitting, it can be assumed that harmalol binds strongly with poly(dG-dC).poly(dG-dC) followed by poly(dA-dT).poly(dA-dT)>poly(dA).poly(dT) and least with poly(dG).poly(dC), indicating hetero GC base pair preference. Thus binding of harmalol shows positive cooperativity in polypurine-polypyrimidine sequences and non cooperativity in alternating purine-pyrimidine sequences. Positive cooperativity has been demonstrated in several DNA-ligand complexes [27]–[31], for example the binding of ethidium, propidium, tilorone and daunomycin to poly(dA).poly(dT) [29], [31]–[33], mitoxantrone to several natural DNAs [34], m-AMSA to calf thymus DNA [35] and isoquinoline alkaloids to natural and synthetic DNA [36], [37]. The cooperative binding has been rationalized as an effect mediated by some conformational change in the helix and it is very interesting to note that such differences in structural/conformational variations are being well differentiated by harmalol. Two similar views are known to explain these effects. Chaires invoked the theory of allosteric interaction developed by Crothers and colleagues [38] to explain the cooperative binding of daunomycin to various polynucleotide structures [27], [39]. According to this model, two structurally different conformations may coexist in the DNA. The binding of the ligand to form I may result in a conformational or allosteric change in the DNA structure to form II. Wilson et al. [29] on the other hand proposed a “preequilibrium” model to explain the unusual binding of propidium to poly(dA).poly(dT), suggesting the initial binding of the antibiotic weakly to an unusual non canonical B-form conformation of the polynucleotide shifting to a more standard B-conformation. It is known that most duplex DNA, natural and synthetic can adopt the gross B-form as defined by their characteristic X-ray patterns. Again among the polynucleotides the maximum cooperativity has been found in poly(dG).poly(dC) and the reason is probably because of the non canonical B-form structure of the polymer [40].

**Table 1:** Comparetive binding parameters for the interaction of harmalol with polynucleotides in 15 mM CP buffer at 25±0.5°C obtained from spectrophotometric and spectrofluorimetric studies^a^.

Polynucleotides	Spectrophotometry (analysis by McGhee-von Hippel)						Spectrofluorimetry φ/φ_o_^b^	Thermal melting ΔT_m_ (°C)
	Binding constant *K_i_* (×10^6^ M^−1^)	n	Cooperativity factor (ω)	*K_i_*ω (×10^6^M^−1^)	Binding mode	Hypochromic effect (%); bathochromic shift (nm)		
poly(dA-dT).poly(dA-dT)	0.43±0.03	0.18	-	-	Non cooperative	33%; 6 nm	1.43	8
poly(dA).poly(dT)	0.02±0.01	0.12	14.0	0.28±0.01	cooperative	21.88%; 4 nm	1.20	6
poly(dG-dC).poly(dG-dC)	4.20±0.05	0.33	-	-	Non cooperative	44%; 13 nm	0.20	ND
poly(dG).poly(dC)	0.002±0.001	0.08	84	0.18±0.01	cooperative	31.36%; 5 nm	0.56	6

ND; not determined.

a Data presented from the average of four determinations in each. Denotes the relative quantum yield value at saturation of nucleotide/alkaloid molar ratio determined as described (ref). ΔT_m_ °C is calculated [T_m_ of complex - T_m_ of duplex polynucleotide] at r_max_.

Thus, together with differences in the stacking arrangement of bases, local structural heterogeneity etc., may give rise to some conformational heterogeneity that may lead to cooperative binding in both the polypurine-polypyrimidine sequences i.e. in homo AT and homo GC sequences, where both the structures have an unusual B-form conformation.

### Fluorescence spectral titration, quantum yield and binding constant analysis

**Figure 4:**
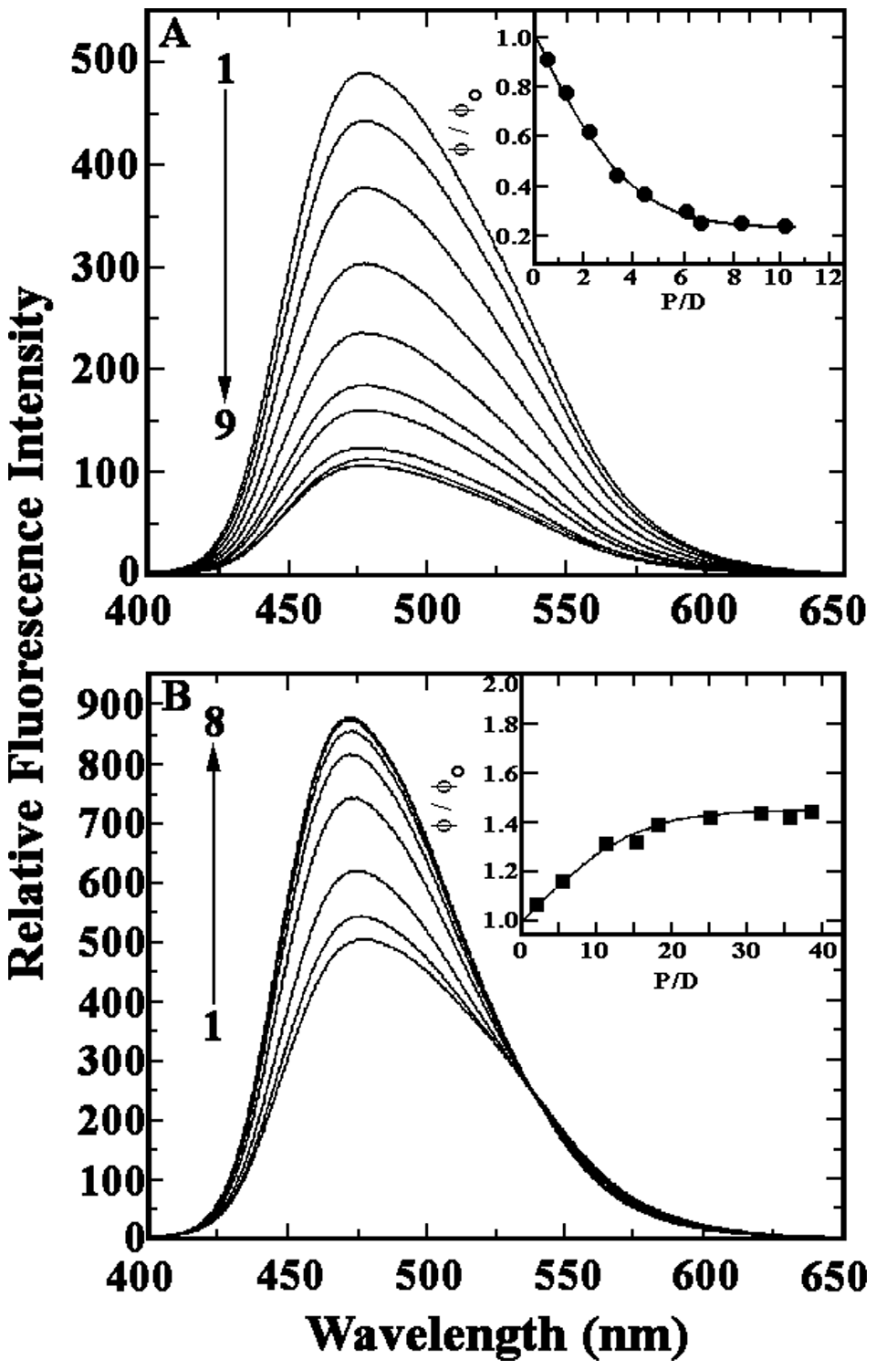
(A) Steady state fluorescence emission spectrum of harmalol (6 µM, Curve 1) treated with various concentrations of poly(dG-dC).poly(dG-dC) in 15 mM CP buffer, pH 6.8 at 25±0.5°C. Curves (2–9) denote 3.50, 7.00, 10.5, 16.80, 20.00, 25.50, 32.40, 37.5 and 42.50 µM of poly(dG-dC).poly(dG-dC). Inset of A: Plots of the relative quantum yield φ/φ_o_ versus P/D for the interaction of harmalol (•-•) with poly(dG-dC).poly(dG-dC). (B) Steady state fluorescence emission spectrum of harmalol (6 µM, Curve 1) treated with various concentrations of poly(dA-dT).poly(dA-dT). Curves (2-8) denote 5.50, 15.60, 25.50, 47.60, 75.0, 100.50 and 128.10 µM of poly(dA-dT).poly(dA-dT). Inset of B: Plots of the relative quantum yield φ/φ_o_ versus P/D for the interaction of harmalol (▪-▪) with poly(dA-dT).poly(dA-dT).

Harmalol was further reported to be a strong fluorophore with an emission spectral peak at 476.8 nm when exited at 376 nm [24]. Fluorescence emission spectra of the alkaloid on titration with the polynucleotides were recorded in the range of 400–650 nm (Figure 4 A,B). Harmalol with hetero GC polymer showed maximum quenching (∼75%) of steady –state fluorescence intensity and attended saturation at a P/D ratio (nucleotide phosphate/alkaloid) of 7.08 (Fig. 4A). Interestingly with both the AT polymers, a gradual enhancement of steady –state fluorescence intensity of harmalol was observed. Harmalol with hetero AT polymer showed ∼35% enhancement of fluorescence intensity with an isoemmisive point at 540 nm and attended saturation at a P/D ratio of 21.35 (Fig. 4B) where as with homo AT polymer it showed ∼25% enhancement with an isoemmisive point at 532 nm and attended saturation at a P/D ratio of 35.20. Isoemissive point was the indication of a clear equilibrium between free and DNA bound form of the alkaloid. Homo GC polymer with harmalol showed least quenching (∼27%) of steady –state fluorescence intensity and attended saturation at a P/D ratio of 41.50. No isoemissive point was observed with the GC polymers. The enhancement or decrease of fluorescence intensity of a fluorophore in the presence of nucleic acids is still controversial. A literature survey revealed that both enhancement and quenching of fluorescence of small molecules in the presence of DNA have been observed and both phenomena have been suggested to be due to strong intercalation [41]. It is likely that intercalation leads to a reduction in the rate of excited state proton transfer to solvent molecules from the alkaloid harmalol, leading to an enhanced fluorescence for the DNA complexes in AT sequences (more with the hetero AT sequence). In the specific case of GC, a reduction has occurred and this fluorophore quenching may be explained by electron sharing/donor properties of the adjacent guanine base. In many cases evidences for the formation of weak or nonfluorescent ground-state complexes between the fluorophores and guanosine residues have been reported. In such complexes, upon complex formation, efficient fluorescence quenching via photoinduced electron transfer can occur [26]. Recently Basu and Kumar have reported that the alkaloid chelethrine also acts as an electron acceptor in the excited state and guanine base acting as the electron donor leading to quenching on binding to hetero GC polynucleotide while with other polynucleotides the fluorescene is enhancing [42]. Furthermore, the position of guanosine in the DNA strand has been reported to be critical for such G-quenching to occur.

The results of fluorescence titration data were converted to the quantum yield of the alkaloid complexes with all the four polynucleotides and presented in Table 1. With the GC polymers the relative quantum yield (φ/φ_o_) of harmalol decreases with increasing P/D values until saturation is reached. Decrease in φ/φ_o_ was more pronounced (Fig. 4A) with harmalol-hetero GC complex compared to harmalol-homo GC complex (figure not shown). Whereas with both the homo and hetero AT polymers (Fig. 4B), φ/φ_o_ of harmalol enhances with P/D and levels off with saturation, latter to a large extent until saturation was achieved. By following the same protocol as in UV spectrophotometry, the results of fluorescence titration data were further analyzed to obtain the Scatchard plot of the binding. The binding constants *K_f_*, stoitiometry *n* and cooperativity factor *ω* were analyzed (figures not shown). Poly(dG-dC).poly(dG-dC) and poly(dA-dT).poly(dA-dT) showed non cooperative binding with a binding constant of 4.60±0.07×10^6^ M^−1^, 0.45±0.04×10^6^ M^−1^, respectively and stoitiomrtry of 0.35 and 0.18, respectively. On the other hand poly(dA).poly(dT) and poly(dG).poly(dC) showed cooperative binding with a binding constant of 0.015±0.001×10^6^ M^−1^, 0.002±0.001×10^6^ M^−1^, respectively, cooperativity factor of 20 and 75, and stoitiomrtry of 0.15 and 0.09, respectively. The values were found to be very close to the UV visible spectrophotometric analysis. Thus the fluorescence data clearly indicates that harmalol binds strongly with hetero GC polymer, followed by hetero AT>homo AT and> homo GC polymers or in other words harmalol prefers hetero GC specific binding.

### UV melting studies

Further, the alkaloid reported to enhance the thermal stability of all the four polynucleotides (figures not shown). Increase in the melting temperature was in the order of about 8°C with the hetero AT polymer and 6°C with both, homo AT and GC polymers, respectively. The melting of the hetero GC polymer under the condition of our experiment was>97°C, so no meaningful data could be deduced in presence of harmalol. It is worth mentioning here that the cooperativity of the thermal melting pattern in all the cases was unaffected in presence of the alkaloid. Thus these data further suggest a strong binding and stabilization of the alkaloid to the polymers but as such no preference for any base pair specificity could be shown by optical melting studies.

### Mode of binding by viscometric analysis

The mode of binding of harmalol to the sonicated polynucleotides was investigated from viscosity studies. The relative specific viscosity of the polymer- alkaloid complex increased sharply as the D/P increased (Fig. S1), suggesting the intercalation of the alkaloid into the helical organization of the sonicated DNA polynucleotides. The relative specific viscosity against D/P for the different DNAs was dependent on the base composition of DNA, being more for the hetero GC polymer, followed by hetero AT polymer>homo AT polymer and lest change with homo GC polymer. For better comparison, the relative specific viscosity of hetero GC polymer - ethidium bromide complexation was also studied with increasing D/P ratios since ethidium is said to be a classical intercalator [43].

### Circular dichroism studies

**Figure 5:**
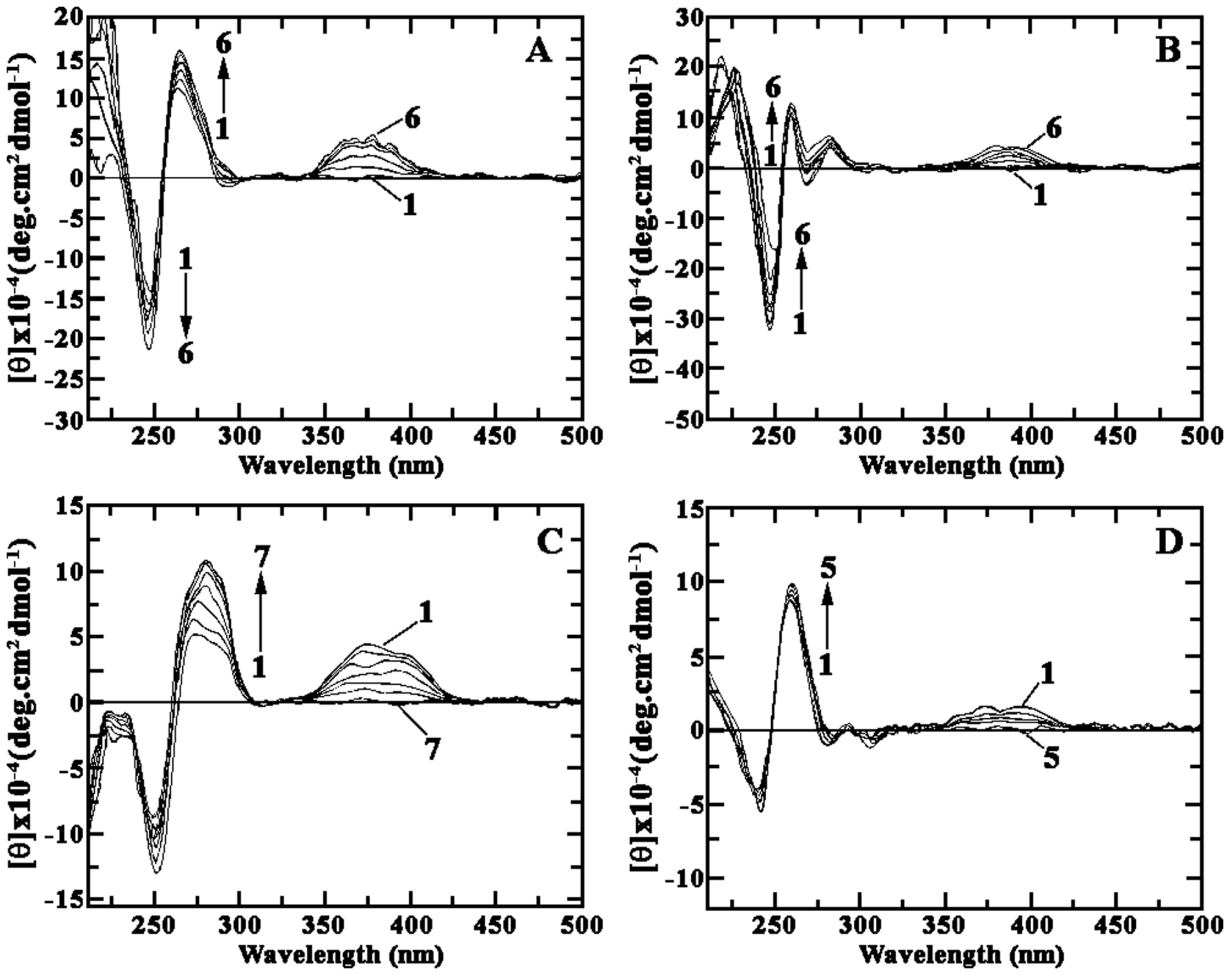
Representative CD spectra resulting from the interaction of harmalol, with the polynucleotides in 15 mM CP buffer of pH 6.8 at 25±0.5°C. (A) Curves (1–6) denote poly(dA-dT).poly(dA-dT) (40 µM) treated with 0, 5.0, 12.0, 15.0, 25.0 and 35.3 µM of harmalol. (B) Curves (1–6) denote poly(dA).poly(dT) (42 µM,) treated with 0, 2.0, 8.0, 12.0, 25.0 and 40.3 µM of harmalol. (C) Curves (1–7) denote poly(dG-dC).poly(dG-dC) (40 µM,) treated with 0, 2.0, 4.0, 8.0, 15.0, 25.0 and 40.3 µM of harmalol. (D) Curves (1–5) denote poly(dG).poly(dC) (45 µM,) treated with 0, 5.0, 16.0, 34.0 and 45.0 µM of harmalol. The expressed molar ellipticity (*θ*) in each case is based on the polynucleotides concentration.

Further, spectropolarimetric or circular dichroism (CD) data provides an independent measure of conformational polymorphism of nucleic acid structures and their interactions with small molecules [44], [45]. CD studies could be understood either through intrinsic CD that depict the changes in the DNA conformation or through induced/extrinsic CD where DNA does not have any contribution and results from the nondegenerative coupling of the ligand chromophore with the transition moments of the adjacent base pairs of the DNA (some chiral electronic interactions π →π* at the binding site) there by giving information about the orientation of the chromophore inside the helical organization. The characteristic CD spectra of all the four polymers studied were remarkably perturbed in presence of harmalol resulting in a rapid increase in the positive band (Fig. 5A–E). The extent of change was more pronounced with hetero GC polymer (Fig. 5C). Interestingly, concomitant with the changes in the intrinsic CD in the UV region (210–310 nm), there appeared an induced CD band in the 300–425 nm regions for the bound alkaloid molecules in all the four polymers, the ellipticity of which increased as the binding progressed. The induced CD band was evidently with more ellipticity in the hetero GC polymer followed by hetero AT, homo AT and homo GC. It is pertinent to note that harmalol is an achiral molecule and is not CD active by itself. Thus, the CD changes revealed that the alkaloid bind differently with the polymers depending on their base sequence, being more strongly with the hetero GC sequence or in other words these results, apart from conformational aspects of the interaction, could be used as a method to show the base pair specificity of the alkaloid.

### Isothermal titration calorimetry (ITC)

**Figure 6:**
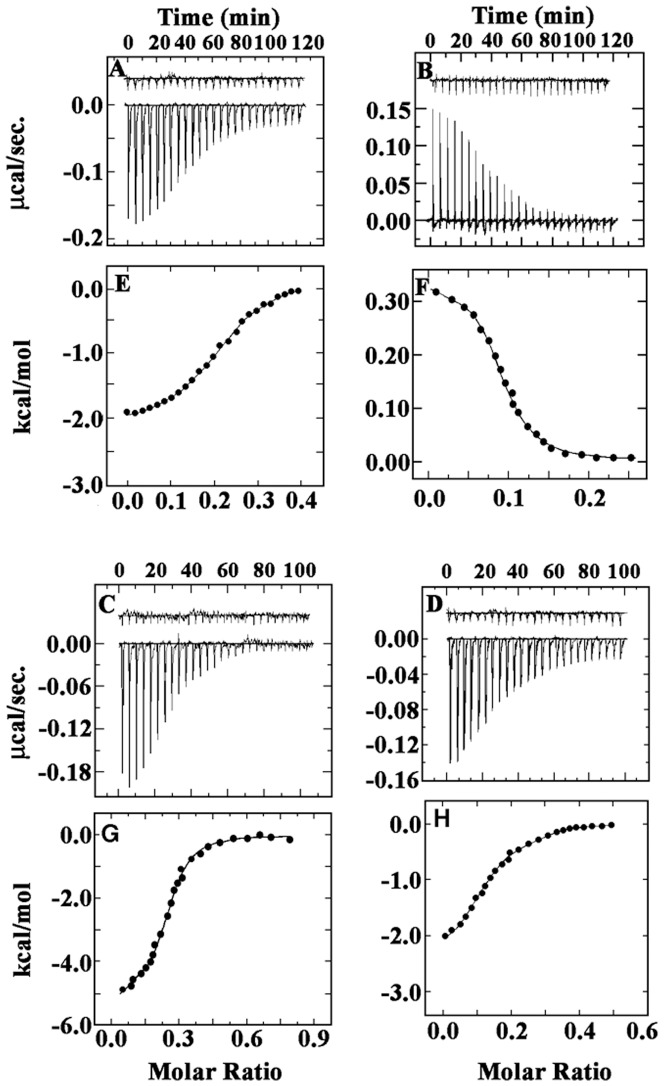
ITC profile for the binding of 1200 µM of (A) poly(dA-dT).poly(dA-dT), (B) poly(dA).poly(dT), (C) poly(dG-dC).poly(dG-dC) and (D) poly(dG).poly(dC) to harmalol (20 µM) at 25±0.5°C, pH 6.8. Each heat burst curve (in the bottom part of upper panel) is the result of a 1.5 µL sequential injection of the polynucleotide into harmalol (curves at the bottom). The top part of upper panel show the heat burst for the injection of the polynucleotide into the same buffer as control in each experiment (curves offset for clarity). The lower panel represent the corresponding normalized heat data against the molar ratio (P/D) (E, F, G and H). The data points (•-•) reflect the experimental injection heats while the solid line represents the calculated best fit of the data. The values of the various thermodynamic parameters obtained are presented in Table 2.

In addition to photophysical data, thermodynamic analysis of drug-DNA binding also provides valuable insights into the nature of the molecular forces that are involved in the complexation. Isothermal titration calorimetry (ITC) is one such sensitive, rapid and reliable methodology for the direct measurement of thermodynamic parameters in various biomolecular interactions [46]–[48]. Since ITC measures heat exchange, it provides a tool independent of the spectroscopic changes that occur in the reaction. In Fig. 6 A–D (upper panels) the raw ITC profiles resulting from the titration of harmalol to the polynucleotide DNAs are presented. Harmalol, due to its aggregation tendency, a reverse protocol has been adopted as reported earlier [24]. Each of the heat burst curve in the figure corresponds to a single injection. The areas under these heat burst curves were determined by integration to yield the associated injection heats. These injection heats were corrected by subtracting the corresponding dilution heats (upper part of the upper panels of Fig. 6 A–D) derived from the injection of identical amounts of the injectants (here it is the respective polunucleotides) into buffer alone. In the lower panel of the figure, the resulting corrected heats were plotted against the respective molar ratios. Here the data points reflect the experimental injection heat while the solid lines reflect calculated fits of data. The corrected isotherms showed only one binding event in all the cases indicating that one type of complexation is formed exclusively, enabling the fitting to a single site protocol in ITC. The binding affinities and the thermodynamic parameters are presented in Table 2. The binding affinity values at 25±0.5°C evaluated from the ITC data are in good agreement with the spectroscopic data (Table 1). The binding is exothermic and predominantly dominated by enthalpy and favorable entropy factor in poly(dG-dC). poly(dG-dC) and poly(dA-dT).poly(dA-dT), respectively, whereas with poly(dA).poly(dT) binding is endothermic and entropy driven and with poly(dG).poly(dC) the binding is entropy driven but unlike homo AT polymer it is exothermic. The Gibbs energy change in each system is more or less similar and in the range of 7–9 kcal/mol. The ITC data of harmalol- poly(dA-dT).poly(dA-dT) complexation (Fig. 6A) yielded a *K*_b_ value of 0.40±0.02×10^5^ M^−1^, an enthalpy change (Δ*H^o^*) of −2.00 kcal/mol and an entropy contribution of (*T*Δ*S^o^*) of 5.69 kcal/mol. The calorimetric data of harmalol- poly(dA).poly(dT) (Fig. 6B) complexation yielded a *K*_b_ value of 0.30±0.02×10^6^ M^−1^, an enthalpy change (Δ*H^o^*) of +0.30 kcal/mol and an entropy contribution of (*T*Δ*S^o^*) of 7.81 kcal/mol and the trend was found to be in good agreement with that reported earlier [29], [33], [37]. Thus in addition to the conformational switch in the polymer on binding as discussed earlier (*vide supra*), a second view has focused on the unusual hydration of poly(dA).poly(dT) and suggested the water release coupled to the binding for the entropically driven drug association [29], [33], [37], [49]. Further a subsequent elegant study by Chaires and coworkers [50] meticulously implicated the involvement of a pre-melting conformational transition from one helical form to another as a physical basis for the unusual thermodynamics of antibiotics binding to this polymer. For the binding with poly(dG-dC).poly(dG-dC) (Fig. 6C), saturated at a polynucleotide/harmalol concentration ratio of 0.4, the ITC data yielded a *K*_b_ value of 3.86±0.07×10^6^ M^−1^, (Δ*H^o^*) of -5.00 kcal/mol and *T*Δ*S^o^* of 4.04 kcal/mol. Further, for the binding with poly(dG).poly(dC) (Fig. 6D), the affinity was 0.22±0.03×10^6^ M^−1^ with an small enthalpy change of −1.98 kcal/mol and a large entropy change factor of 5.35 kcal/mol. Thus from the above binding thermograms it is again very clearly indicating, that harmalol shows maximum preference for hetero GC polymer followed by hetero AT>homo AT and least binding with homo GC polymer.

**Table 2:** ITC derived binding and thermodynamic profiles for the interaction of harmalol to various polynucleotides^a^.

Polynucleotides	*K*_b_ (×10^6^ M^−1^)	n	Δ*G^o^* (kcal/mol)	Δ*H^o^* (kcal/mol)	*T*Δ*S^o^* (kcal/mol)
poly(dA-dT).poly(dA-dT)	0.44±0.02	0.18	−7.74	−2.00	5.74
poly(dA).poly(dT)	0.30±0.02	0.11	−7.51	+0.30	7.81
poly(dG-dC).poly(dG-dC)	3.86±0.07	0.30	−9.04	−5.00	4.04
poly(dG).poly(dC)	0.22±0.03	0.12	−7.33	−1.98	5.35

a Average of three determinations in CP buffer of 15 mM [Na^+^], pH 6.8. Values of Δ*G^o^* were determined using the equation Δ*G^o^* = −*RT ln K*_b_ and *T*Δ*S^o^* = Δ*H^o^*−Δ*G^o^*. n denotes the binding site size.

### Heat capacity change of binding

**Figure 7:**
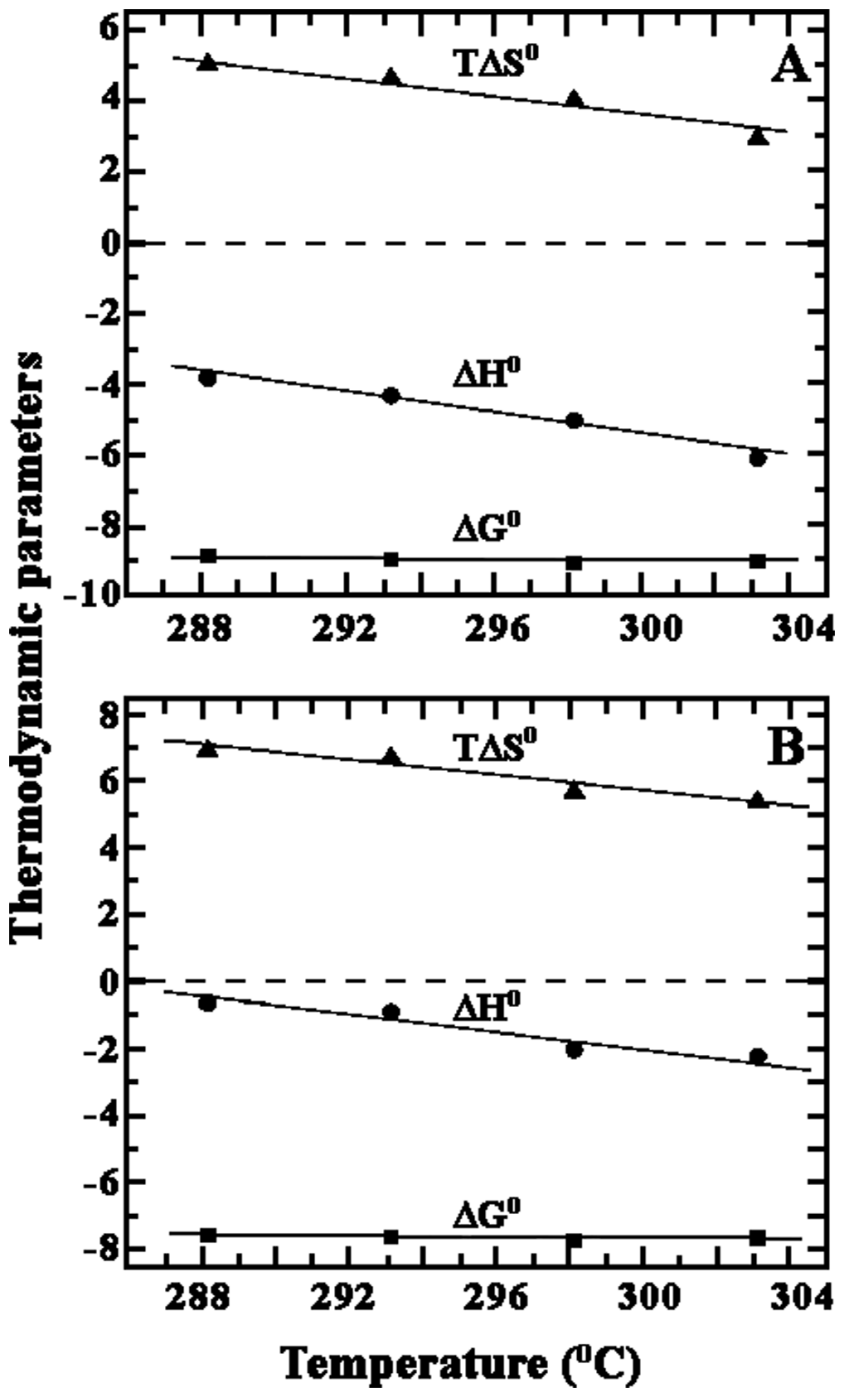
Temperature dependence of the thermodynamic parameters, *T*Δ*S*°, Δ*H*° and Δ*G*° for the binding of harmalol to (A) poly(dG-dC).poly(dG-dC) (B) poly(dA-dT).poly(dA-dT). Values of all the parameters are presented in Table 3.

We have also studied the temperature dependence of the binding of harmalol with poly(dA-dT).poly(dA-dT) and poly(dG-dC).poly(dG-dC) and determined the heat capacity values using the standard relationship, Δ*C_p_^o^* = δ(Δ*H*)/δT. This parameter provides a mean for linking structural and energetic data and should describe the hydration -dehydration effects that occur during the binding process. Studies were performed in the range of 15–30°C and the thermodynamic parameters elucidated are presented in Table 3. The association constant for harmalol binding to poly(dA-dT).poly(dA-dT) varied from 0.52±0.09×10^6^ M^−1^ at 15°C to 0.31±0.01×10^6^ M^−1^ at 30°C (Table 3). The interaction was overwhelmingly entropy driven at all the temperatures, but binding enthalpy gradually increased and the entropy terms decreased despite increase in temperature. Interestingly, the Gibbs energy exhibited only small changes (varied from −7.58 to −7.66 kcal/mol). The binding affinity of harmalol- poly(dG-dC).poly(dG-dC) complexation on the other hand varied from 4.70±0.47 ×10^6^ M^−1^ at 15°C to 2.96±0.0.06 ×10^6^ M^−1^ at 30°C. Gibbs energy exhibited only small changes, varied from −8.85 to −9.03 kcal/mol. The binding enthalpy increased and the entropy term (a favorable term to the Gibbs energy) decreased with increasing temperature keeping Δ*G^o^* almost constant. The temperature dependence of the enthalpy yielded an estimate for heat capacity (Δ*C_p_^o^*). The reaction enthalpy and entropy both of which were strong functions of temperature compensate (Δ*H^o^* and TΔ*S^o^* are parallel) to make the reaction Gibbs energy almost independent of temperature. Such compensation was observed for many biomolecular interactions and the phenomena were suggested to be due to a significant hydrophobic component to the binding energies [51]. The slope of the line revealed values of −0.150 and −0.118 kcal/mol K for harmalol binding to poly(dG-dC).poly(dG-dC)and poly(dA-dT).poly(dA-dT), respectively (Fig. 7A,B). Similar heat capacity values have been observed from ITC data for a variety of small molecules binding to DNA [51]–[54]. It is reported that for intercalators or planar molecules a large hydrophobic contribution to the binding Gibbs energy is expected due to their aromatic ring system and binding should be energetically favorable [55]. From the Records expression [55], Δ*G*_hyd_^o^ = 80 (±10)×Δ*C_p_^o^*, the Gibbs energy contribution to the hydrophobic transfer step of the ligand binding may be calculated. Hence, values of Δ*G*_hyd_^o^ for harmalol binding to poly(dA-dT).poly(dA-dT) and poly(dG-dC).poly(dG-dC) (Table 3) was calculated to be −9.4 and −12.0/kcal mol^−1^ respectively (Fig. 7A,B). Though, Δ*G*_hyd_^o^ value for harmalol binding to poly(dA-dT).poly(dA-dT) is slightly lower, but with poly(dG-dC).poly(dG-dC), the value was well within the range that was observed for classical intercalators [51], [54], [56]. Thus these results clearly indicate the involvement of a remarkably large hydrophobic contribution in harmalol-polynucleotide interaction.

**Table 3:** Temperature dependent thermodynamic parameters for the binding of harmalol to the hetero polynucleotides.

Polynucleotides	Temperature (K)	*K*_b_ (×10^6^ M^−1^)	Δ*G^o^* kcal/mol	Δ*H^o^* kcal/mol	*T*Δ*S^o^* kcal/mol	Δ*C_p_* ^o^ kcal/mol K	Δ*G_hyd_* kcal/mol
poly(dG-dC).poly(dG-dC)	288.15	4.70±0.47	−8.85	−3.8	5.05		
	293.15	4.20±0.27	−8.94	−4.30	4.64	−0.150	−12.0
	298.15	3.86±0.07	−9.04	−5.00	4.04		
	303.15	2.96±0.06	−9.03	−6.07	2.96		
poly(dA-dT).poly(dA-dT)	288.15	0.52±0.09	−7.58	−0.61	6.97		
	293.15	0.45±0.07	−7.63	−0.87	6.76	−0.118	−9.4
	298.15	0.44±0.02	−7.74	−2.00	5.74		
	303.15	0.31±0.01	−7.66	−2.20	5.46		

All the data in this table are derived from ITC experiments conducted in 15 mM CP buffer, pH 6.8 and are average of three determinations. *Kb* and Δ*H* o values were determined from ITC profiles fitting to Origin 7 software as described in the text. The values of Δ*Go* and *T*Δ*So* were determined using the equations Δ*Go* = −RTln*K*b and *T*Δ*So* = Δ*Ho*−Δ*Go*. All the ITC profiles were fit to a model of single binding sites.

### Molecular docking analysis

**Figure 8:**
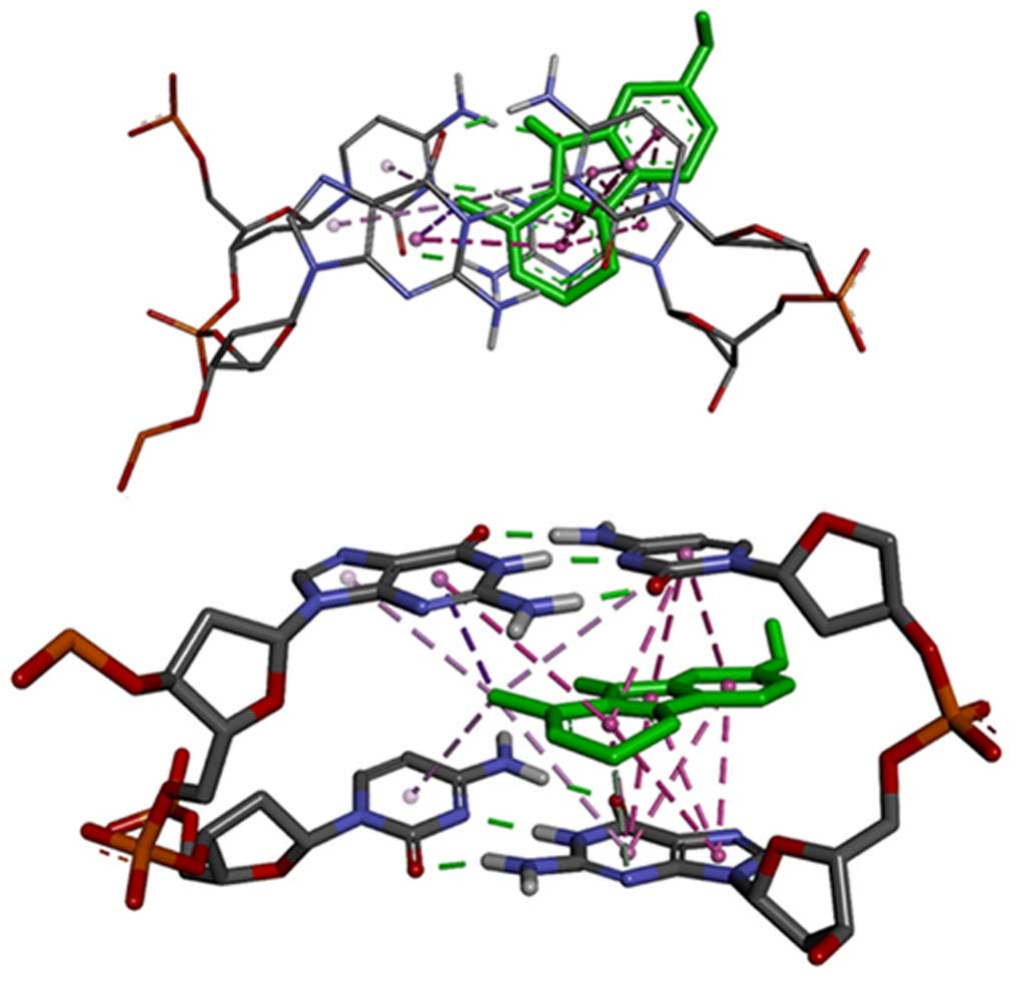
Harmalol binding with intercalation cavity at 5′-CpG-3′ of duplex DNA (PDB ID 4BZV). Right image shows harmalol in green color. Magenta colored lines indicate hydrophobic forces of interaction.

The experimental results obtained so far have clearly suggested that Harmalol molecule at pH 6.8 interacts with DNA through intercalation mode, more preferably and strongly with hetero GC sequence than the AT sequences. In order to model the intercalation mechanism of harmalol, it is essential to obtain a sizeable intercalation cavity to acquire reasonable estimates of binding affinity and ligand poses in docking calculations. Previously Xio *et al*. had also performed the molecular modelling by using Biosyn modelling package to show the binding of beta carboline derivatives to DNA sequences [20]. The structures containing intercalation cavities are explored in the PDB (Protein Data Bank). Presently two DNA sequences, 4BZV and 1G3X, have been selected for the docking analysis. A DNA-ligand complex 4BZV has been reported to contain intercalation cavity at 5′-CpG-3′ site while the complex 1G3X contain intercalation site at 5′-ApA-3′. Molecular docking calculations produced 9 binding poses at CpG binding site while in the case of ApA binding site, 20 binding poses were obtained in the range of 2 kcal/mol. Docking also produced binding affinity values that are close to the experimental values obtained from fluorescence and ITC experiments. In the case of harmalol-DNA docking, both the DNA sequences, 4BZV and 1G3X, gave binding Gibbs energy values of −8.62 kcal/mol and −7.41 kcal/mol, respectively. Further, these Gibbs energy values were used to calculate binding constant values of 1.84×10^6^ M^−1^ and 0.25×10^6^ M^−1^, respectively using the equation, Δ*G^o^* = −RTln*K*_b_, where ΔG^o^ is binding Gibb's energy in kcal/mol, R is gas constant and T is temperature in kelvin. When analyzing the binding interaction, it is essential to consider the structure of the ligand molecule first in order to establish the sources of intermolecular interaction forces present in the ligand. The primary purpose of docking calculations is to understand the structure of binding poses of drugs in receptor bound states that match with the experimental observations. Normally, docking produces several bound poses in a single calculation. Since docking poses and their estimated binding energy does not have direct relation with the experimental binding constants or affinity values, it is imperative to use appropriate search algorithms and scoring functions to obtain the values as close to the experimental observations as possible. This implies that in the docking results, those poses that are closer in experimental affinity values should be more closely analyzed. Typically, the best estimates of binding free energy values in docking are those that come closer to the experimental values [57], [58]. The difference, however, between the two values can be attributed to several factors such as choice of calculation program, solvent and ionic environment considerations, etc. In reality, the exact match of experimental and computational affinities in terms of numbers is extremely difficult. However, in the case of a number of compounds, a trend can be obtained which provides for the reasonable comparison of affinity values. It is clear from the structure of harmalol (*vide supra*, Fig. 1) that there is a strong possibility for a significant contribution from the hydrophobic forces (pi-pi stacking, pi-alkyl group interactions, etc) in the overall binding affinity values between harmalol and DNA bases. The analysis of binding poses of harmalol with both the DNA sequences reveals that there is indeed a strong hydrophobic force that contributes in the overall binding (Fig. 8). Besides the hydrophobic forces, the H-bonding was also observed, though to a lesser extent. Out of the 9 bound poses obtained from modified DNA 4BZV, only five poses were found to contain H-bonds. On the other hand, 20 poses obtained from modified DNA 1G3X, only 4 contained H-bonds. This shows that although the harmalol contains potential H-bond donor and acceptor groups, their significance in the DNA bound state is limited as compared to stacking forces. Further, the actual H-bonds observed in both the cases of docking, were between DNA bases and two ringed Nitrogens. The poses were oriented in such a way that H-bonds between DNA bases and oxygen atom of Harmalol was observed only rarely. Finally, the absence of any side chain of significant length does not provide for the anchoring of harmalol inside the minor groove of DNA as observed in the case of other molecules [57], [58].

### Cell viability test by MTT assay

**Figure 9:**
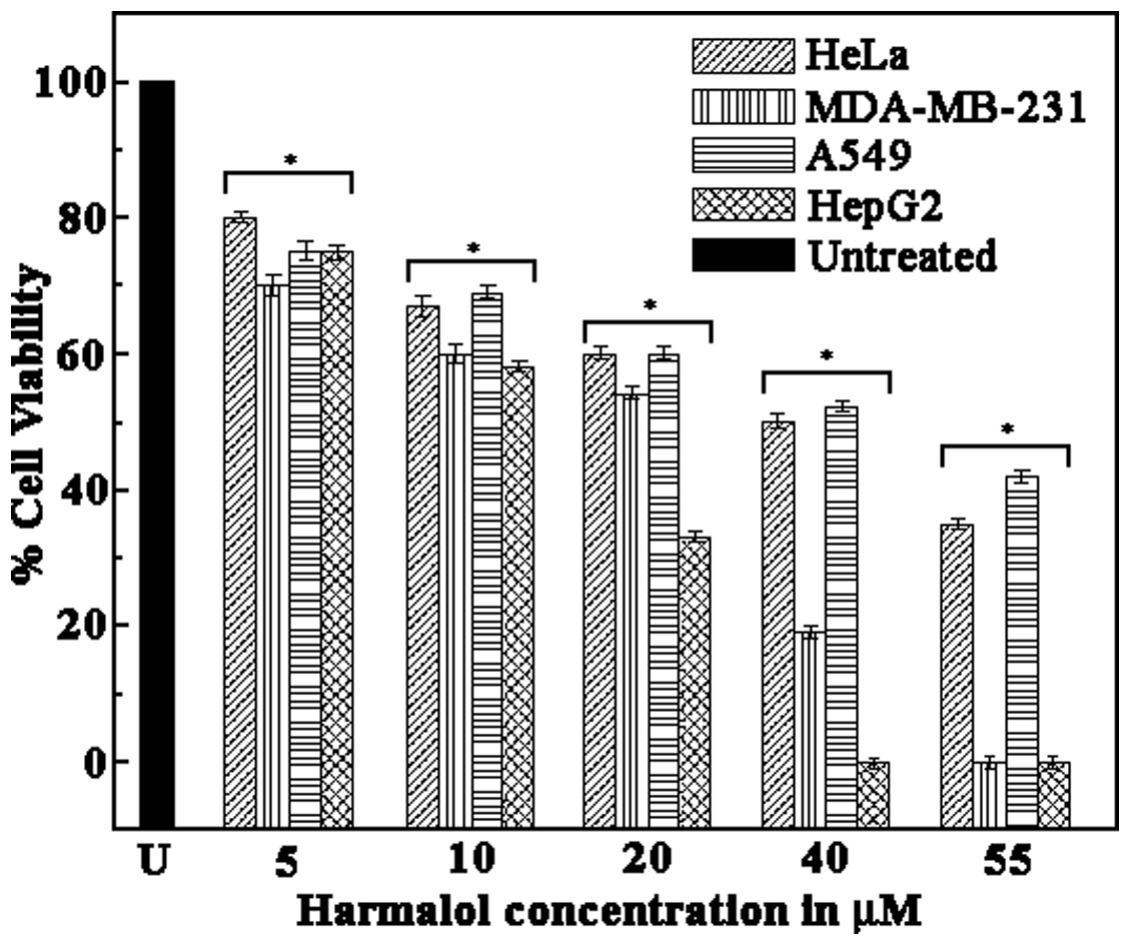
Effect of varying concentrations (5, 10, 20, 40 and 55 µM) of Harmalol for 72 hours on the viability (MTT) of different cancer cells (HeLa, MDA-MB-231, A549 and HepG2) resulted in a significant dose-dependent reduction in the viability of the cells. The data are represented as the means ± SEM of three independent experiments. Significant values are calculated against untreated control cells and analyzed with ANOVA test. (*P<0.05 vs. untreated).

Binding studies of the natural alkaloid with DNA will be always incomplete without the biological interpretation and importance of that alkaloid, hence cytotoxicity of harmalol against various human cancer cell lines, is one of the important aspect that further signifies the anticancer activity of the ligand. Cytotoxicity of harmalol was determined by treating different cancer cell lines viz. HeLa, MDA-MB-231, A549 and HepG2 with various concentrations of the alkaloid (5, 10, 20, 40 and 55 µM) at 37°C for 24, 48 and 72 hrs followed by MTT assay (Fig. 9). The dose dependent reaction in the viable cells was observed and IC_50_ values were calculated and presented in Table 4. HepG2 was reported to have the least IC_50_ value of 14 µM followed by MDA-MB-231 with IC_50_ value of 24 µM, HeLa with IC_50_ value of 42 µM, and A549 with the maximum IC_50_ value of 45 µM. The data are presented as the means ± SEM of three independent experiments. Significant values are calculated against untreated control cells and analyzed with ANOVA test. (P<0.05 *vs* untreated). Thus the technique indicates that harmalol is most effective against HepG2 cell line and least effective against A549. Previously also harmalol has been reported to be very effective on dioxin mediated induction of CYP1A1 in human hepatoma HepG2 cells [59]. It has been further postulated that it's antimutagenic and antigenotoxic effects in mammalian cells is because of the antioxidant properties and the antioxidant properties is mainly due to the stabilization of the formed radicals by the resonance structures [60].

**Table 4:** *In vitro* a cytotoxicity of harmalol (IC_50_ b µM).

Cancer cell lines	72 hrs incubation with the alkaloid (in µM)
HeLa^c^	42
MDA-MB-231^c^	24
A549^c^	45
HepG2^c^	14

a Data represent the mean values of three independent determinations.

b Cytotoxicity as IC_50_ for cell line is the concentration of compound which reduced by 50% the optical density of treated cells with respect to untreated cells using MTT assay.

c Cell lines include non small cell cervical carcinoma (Hela), breast carcinoma (MDA-MB-231), lung carcinoma (A549) and liver carcinoma (HepG2).

This method is particularly convenient for the rapid assay of replicate cell cultures in multi-well plates. Plate readers are capable of measuring the absorbance of each well of a standard 96-well plate. The basic principle is that the tetrazolium dye, MTT, is cleaved to a colored product by the activity of dehydrogenase enzymes and this indicates high levels of mitochondrial activity in the viable cells. The color development (purple) is proportional to the number of metabolically active cells. However there is considerable variation in results between cell lines with some cells producing a very low response. Cells with a significant level of oxidative metabolism such as CHO, reported to produce a good response in the MTT assay [61].

## Conclusions

The present study investigate the interaction of beta carboline alkaloid, harmalol with polynucleotides of different base sequences and reveal the specificity and mode of binding in terms of structural differences and correlate the energetics and cytotoxicity of the interaction. Comparative studies unequivocally established that harmalol binds strongly with hetero GC polymer followed by hetero AT polymer by mechanism of intercalation and showed maximum cytotoxicity on HepG2 with IC_50_ of 14 µM. Further the thermodynamic profiles showed that the binding of the alkaloid was predominantly a single site enthalpy driven exothermic binding with a hydrophobic contribution to the binding Gibbs energy with hetero GC and AT polynucleotides. The analysis of binding poses of harmalol with both the DNA sequences from molecular docking analysis also revealed a strong hydrophobic force that contributes in the overall binding. With homo AT and homo GC polynucleotides, binding have been reported to be overwhelmingly entropy driven and this has been rationalized by the release of more solvent coupled with a mandatory conformational change in the polymer induced by drug binding because of the non-canonical B-form rigid conformation of the polymers. Thus, the results highlighted the importance of structural elements in small molecule in stabilizing the DNA structure for developing better therapeutic agents.

## Materials and Methods

### Biochemicals

Harmalol as a hydrochloride salt was obtained from Sigma-Aldrich (St. Louis, MO, USA). The purity of the sample was confirmed by thin layer chromatography, melting point determination and NMR spectroscopy. Harmalol was dissolved in 15 mM citrate-phosphate buffer of pH 6.8 at 42–45°C and its concentration was determined using molar extinction coefficient value of 19,000 M^−1^ cm^−1^, calculated by us, at 371 nm. However, according to Biondic and Balsells, the molar extinction coefficient value of harmalol in various organic solvent media were reported to be between 18,000–22,000 M^−1^ cm^−1^ [62]. Further, Alomer *et al.* have also commented on the ε value for the acidic species of this alkaloid at 371 nm to be 15,904 M^−1^ cm^−1^ [63].

Synthetic polynucleotides, poly(dA).poly(dT), poly(dA-dT).poly(dA-dT), poly(dG).poly(dC) and poly(dG-dC).poly(dG-dC) were also obtained from Sigma-Aldrich Corporation (St. Louis, MO, USA) and used as such. Each polynucleotide concentration in terms of nucleotide phosphate was determined spectrophotometrically using molar extinction coefficient values reported previously [64].

### Cell lines and culture conditions

For the experiments, four types of human cancer cell lines *viz.* HeLa (cervix epitheloid carcinoma), MDA-MB-231 (breast epitheloid carcinoma), A549 (lung epitheloid carcinoma) and HepG2 (liver epitheloid carcinoma) were chosen. All the cell lines were obtained from National Centre for Cell Science, Department of Biotechnology, Govt. of India, Pune. Cells were grown in DMEM with 10% FBS and 1% Antibiotic and Antimycotic solution in a CO_2_ air-jacketed incubator (ESCO, celculture CO_2_ INCUBATOR, CCL-170T-8-UV) at 37°C in a humidified atmosphere of 5% CO_2_ and 95% air during 3–5 days. Then the cells were washed twice with phosphate-buffered saline (PBS) at pH 7, then trypsinized (0.05% gibco) and incubated during 3–5 min; 10 ml DMEM containing 10% FBS was added, the cells were resuspended and quantified in Neubauer chamber.

### Cell viability test: MTT assay

We tested the percentage of cell viability from the above mentioned cell lines by MTT assay [65]. MTT reagent (1 mg/ml of the tetrazolium dye and 3-(4,5-dimethylthiazol-2-yl)-2,5-diphenyltetrazolium bromide dissolved in phosphate buffer saline, pH 7.4) was obtained from SRL, Pvt. Ltd. We quantified the data of MTT assay by using microplate ELISA reader (Multiscan EX Thermo Electron, Corporation, USA) [66]. Briefly, cells were seeded in 96 well plates at a density of 2×10^3^ cells/well and treated with harmalol of various concentrations (5, 10, 20, 40, and 55 µM) at 37°C for 72 hrs. After the exposure period, 10 µl of MTT (1 mg/ml) was added into each well and incubated at 37°C for 4 hrs. Excess media and MTT were removed. The remaining MTT-formazan crystal was dissolved in 100 µl DMSO (dimethyl sulphoxide). The purple crystals of Formazan formed which were proportional to viable cells. The color absorbance of each well was recorded at 595 nm.

### Preparation of buffer

Experiments were carried out in 15 mM Citrate-Phosphate (CP) buffer of pH 6.8. The buffer solution was passed through 0.45 µm syringe filters (Millipore India Pvt. Ltd. Bangalore, India) to remove any particulate matter. MilliQ water was used throughout. All other reagents and chemicals were of analytical grade purity.

### Competition dialysis assay

Competition dialysis assay is an effective tool developed by Chaires *et al.* (1999) [25], [26] based on the fundamental thermodynamic principle of equilibrium dialysis for the determination of binding of small molecules that interact to nucleic acids with structural and sequence selectivity. Briefly, 200 mL of the alkaloid of 1 µM concentration (C_f_, dialysate solution) was placed in a beaker, 0.5 mL of each of the polynucleotide samples of 150 µM (S_total_) in nucleotide -phosphate unit were pipetted into separate 0.5 mL Spectra/Por Dispo Dialyzer unit (Spectrum Laboratories, Inc., CA, USA). All the dialysis units were then placed in a beaker containing the dialysate solution and allowed to equilibrate with continuous slow stirring for 24 hrs at 25±0.5°C. The beaker was covered with parafilm and wrapped in foil. At the end of the equilibration period, the samples were carefully transferred to microfuge tubes, and were taken to a final concentration of 1% (w/v) sodium dodecyle sulphate (SDS) by the addition of appropriate volumes of a 10% (w/v) stock solution. The total concentration of the alkaloid (C_t_) within each dialysis unit was then determined spectrophotometrically by measuring the OD at λ_max_ i.e. at 371 nm. The amount of bound alkaloid (C_b_) was determined by the difference (C_b_ = Ct−C_f_). Data were plotted as a bar graph using Origin 7.0 software (MicroCal, Inc., CA, USA). This data was then used to calculate the apparent binding constant (K_app_) using the relation [25], [26]

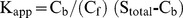
(1)


### Absorbance and fluorescence spectral studies

Absorbance spectra were measured on a Jasco V-630 double beam monochromator spectrophotometer (Jasco International Co. Ltd. Tokyo, Japan) equipped with a thermoelectrically controlled cell holder in matched quartz cells of 1 cm path length under stirring at 25±0.5°C. Steady state fluorescence measurements were performed on a Hitachi F4010 fluorescence spectrometer (Hitachi Ltd., Tokyo, Japan) in fluorescence free quartz cells of 1 cm path length. The excitation wavelength for harmalol hydrochloride was 376 nm [24]. All measurements were done under conditions of stirring keeping excitation and emission band passes of 2.5 and 10 nm, respectively. The sample temperature was maintained at 25±0.5°C.

### Analysis of the binding affinity by Scatchard plots

In alkaloid-polynucleotide titration experiment, the amount of free and bound harmalol was determined following the methodology described by Chaires *et al.* [67]. In absorbance, following each addition of the alkaloid to the polymer solution (40 µM), the respective isosbestic point *viz.* 407 nm with poly(dG-dC).poly(dG-dC), 412 nm with poly(dG).poly(dC), 409 nm with poly(dA-dT).poly(dA-dT) and 425 nm with poly(dA).poly(dT), the total drug concentration (C_t_) present was calculated as C_t_ = A_iso_/ε_max_. This quantity was used to calculate the expected absorbance (A_exp_) at wavelength maximum, A_exp_ = C_t_ ε_max_. The difference in A_exp_ and the observed absorbance (A_obsd_) was then used to calculate the amount of bound alkaloid as C_b_ = A/Δε = (A_exp_−A_obsd_)/(ε_f_−ε_b_). The amount of free alkaloid concentration was determined by the difference, C_f_ = C_t_−C_b_. The molar extinction coefficient of the completely bound alkaloid in each case was determined by adding a known quantity of the alkaloid to a large excess of DNA polynucleotide and on the assumption of total binding, ε_b_ = A_max_/C_t_. Alternatively, the absorbance of a known quantity of the alkaloid was monitored at the wavelength maximum λ_max_ while adding known amounts of the polynucleotide until no further change was observed. The wavelength of the isosbestic point was also determined from these mixing experiments. Both these protocols gave similar values within experimental errors. In fluorescence C_b_ was calculated from the relation C_b_ = C_t_(*I−I_o_*)/(*Vo−1*)*I_o_*, where C_t_ is the known total alkaloid concentration, *I* is the observed fluorescence, *I_o_* is the fluorescence intensity of the identical concentration of alkaloid in the absence of polynucleotides and *V_o_* is the experimentally determined ratio of the fluorescence intensity of totally bound alkaloid to that of free alkaloid. Binding data obtained from spectrophotometric and spectrofluorimetric titration was cast into Scatchard plots of r/C_f_ versus r, where the binding ratio r is defined as, r = C_b_/[DNA]_total_. Scatchard plot was analyzed for cooperative and non cooperative binding using the following equations of McGhee and von Hippel [68]

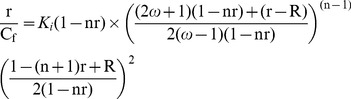
(2)

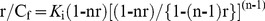
(3)respectively, where, R = {[1−(n+1) r]^2^+4ω r (1−nr)}^1/2^, *K_i_* is the intrinsic binding constant to an isolated binding site, *n* is the number of base pairs excluded by the binding of a single ligand molecule and *ω* is cooperative factor. The binding data were analyzed using the Origin 7.0 (Origin Lab Corporation, Southampton, MA, USA) software to determine the best-fit parameters to Eq. (1) and (2).

### Determination of quantum yield by fluorescence spectroscopy

Quantum yield calculations were made according to the equation of Parker and Rees (1960) as described earlier. [69]

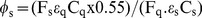
(4)where F is the integrated area of the fluorescence emission curve in arbitrary unit, ε represents the molar extinction coefficient and C represents the molar concentration of sample (s) and quinine sulphate (q) respectively. Quinine sulphate in 0.1 N H_2_SO_4_ was utilized as reference standard for quantum yield measurements.

### UV melting measurements

Melting curves were recorded on a Jasco V- 630 unit equipped with the peltier controlled Jasco PAC-743 model accessory (Jasco International Co. Ltd. Tokyo, Japan) as described earlier [24]. In a typical experiment, the polymer sample (20 µM) was mixed with varying concentrations of the drug in the degassed buffer in the micro optical cuvettes of 10 cm path length and the temperature of the microcell accessory was raised at a heating rate of 0.5°C/minutes while continuously monitoring the absorbance change at 260 nm. Melting curves gave the melting temperature, *T_m_*, the midpoint temperature of the drug bound polymer unfolding process and are average of three experiments.

### Solution viscometric study

For viscometric experiments, samples of linear duplex polynucleotides were sonicated in a Labsonic 2000 sonicator (B. Braun, Swiss) by using a needle probe as described earlier [36],[69]. After sonication, DNA samples were extensively dialyzed against the buffer under sterile conditions. Viscosity measurements revealed that the polynucleotide samples after sonication had an average size of 270±40 base pairs. A Cannon-Manning Type 75 semimicro viscometer mounted vertically in a constant temperature bath (Cannon Instruments Co., State College, PA, USA) maintained at 25±0.5°C as described previously was used for viscosity studies. Flow times were measured using an electronic stopwatch model HS-30W (Casio Computer Co. Ltd., Japan) with an accuracy of ±0.01 s. Relative viscosities for DNA in either the presence or absence of the alkaloids were calculated from the relation 
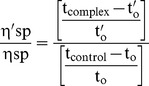
(5)where, η′_sp_ and η_sp_ are specific viscosities of the alkaloid-nucleic acid complex and the nucleic acid respectively, t_complex_, t_control_, t′_o_ and t_o_ are the average flow times for the complex, free nucleic acid, solvent for the complex and solvent for the free nucleic acid respectively.

### Molecular docking

Rigid body molecular docking calculations were accomplished using AutoDock-vina program (version 1.1.2) from the Scripps Research Institute [70]. Autodock-vina performs faster and more accurate docking calculations than autodock [71]–[73]. Autodock-Vina has been used earlier for ligand-DNA docking studies [58]. In rigid-body docking method, the DNA structure remains rigid while the ligand remains flexible. This enables the ligand to explore possible binding poses within a specified area on the receptor. To utilize the intercalation cavity in both the PDB files, the original ligands contained in the PDB files were removed manually using MOE (molecular operating environment) program. The resulting DNA structures were then energy minimized in MOE program by using AMBER99 force field with a tether weight of 10 on heavy atoms to obtain modified DNA duplexes having an empty intercalation cavity. Before docking, the receptor and ligand coordinate files were converted into PDBQT format using MGLTools (version 1.5.4). For docking calculations with the modified 4BZV, a 3-dimensional grid box of 28 × 30 × 28 was prepared with a grid spacing of 0.375 Å. The grid was centered on coordinates x = 28.929, y = 33.513, z = 34.682. In the case of the modified 1G3X, the grid box dimension was 38 × 24 × 48 and the grid box center was at x = 57.642, y = 52.855, z = 58.635 with a grid spacing of 0.375 Å. For each docking calculation, 20 different poses were requested within the energy range of 2 kcal/mol. Due to the large search space volume, the exhaustiveness of the calculation was increased to 16 which is the 2 times the default value in autodock-vina. All other parameters were kept at their default values. The analysis and representation of docked poses were performed using Discovery Studio Visualizer from Accelrys Inc. and UCSF Chimera programs.

### Circular dichroism studies

Circular dichroism (CD) spectra were recorded on a Jasco J815 spectropolarimeter (Jasco International Co. Ltd.) attached with a temperature controller and temperature programmer (model PFD 425 L/15) interfaced to a PC. For monitoring the conformational changes in the CD (210–500 nm regions) a constant concentration of the DNA sample (65 µM) was titrated with increasing concentration of the alkaloids in rectangular quartz cell of 1 cm path length. The molar ellipticity values [θ] were expressed in terms of per nucleotide phosphate (210–500 nm regions).

### Isothermal titration calorimetry

Isothermal titration calorimetry (ITC) experiments were performed on a GE Microcal ITC 200, (Northampton, USA) microcalorimeter as described earlier [24]. Origin 7.0 software was used for data acquisition and manipulation. ITC data provided *K_b_*, the binding constant, Δ*H^o^*, enthalpy change and Δ*S^o^*, the entropy change values. Each experiment was repeated three times and the error value that reflects the standard deviations among the different runs was always less that 10%.

## Supporting Information

Figure S1 **A plot of change of relative specific viscosity of (•-•) poly(dG-dC).poly(dG-dC), (▪-▪) poly(dA-dT).poly(dA-dT), (□-□) poly(dA).poly(dT) and (○-○) poly(dG).poly(dC) with increasing concentration of harmalol in 15 mM CP buffer, pH 6.8 at 25±0.5°C.** The specific viscosity was calculated from equation (5) described in section 2.9. The concentration of each of the polynucleotide was 450 µM, respectively.
(TIF)
