# Supplementary figures and images for: Sequence Specific Binding of Beta Carboline Alkaloid Harmalol with Deoxyribonucleotides: Binding Heterogeneity, Conformational, Thermodynamic and Cytotoxic Aspects
Source: PLoS One. 2014 Sep 23;9(9):e108022. doi: 10.1371/journal.pone.0108022 (PMC4172587; doi:10.1371/journal.pone.0108022)

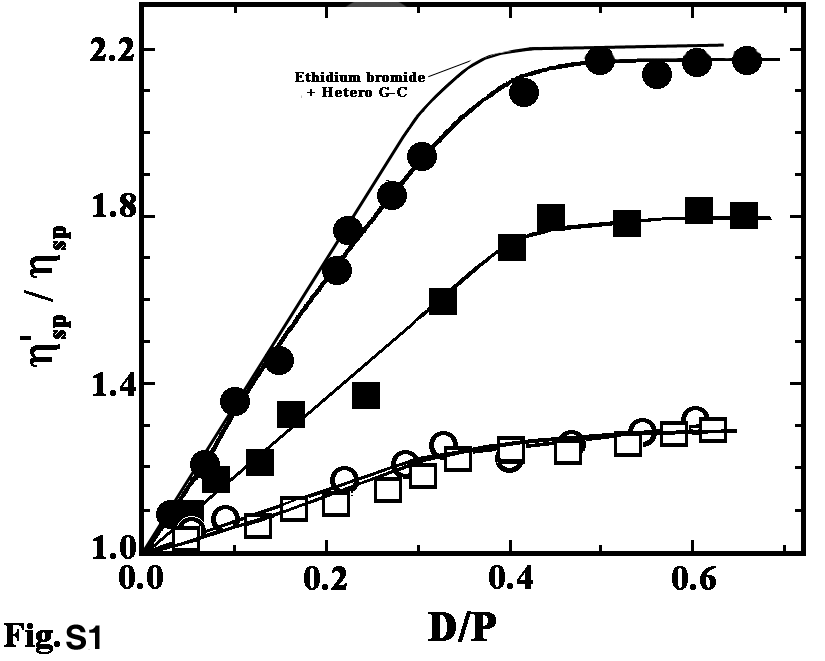

Supplement: Figure S1 — A plot of change of relative specific viscosity of (•-•) poly(dG-dC).poly(dG-dC), (▪-▪) poly(dA-dT).poly(dA-dT), (□-□) poly(dA).poly(dT) and (○-○) poly(dG).poly(dC) with increasing concentration of harmalol in 15 mM CP buffer, pH 6.8 at 25±0.5°C. The specific viscosity was calculated from equation (5) described in section 2.9. The concentration of each of the polynucleotide was 450 µM, respectively. (TIF) [file pone.0108022.s001.tif]
